# Supplementary material for: The p75 neurotrophin receptor in AgRP neurons is necessary for homeostatic feeding and food anticipation
Source: eLife. 2020 Jan 29;9:e52623. doi: 10.7554/eLife.52623 (PMC7056271; doi:10.7554/eLife.52623)
Supplement: Table 1—source data 1. [file elife-52623-table1-data1.docx]

**Table 1- source data 1**

| ***p* values** | |  |  |
| --- | --- | --- | --- |
| **ZT4** |  |  |  |
| *Body Weight* | WT-KO | Fed, p=0.037 | Fasted, p=0.247 |
|  | Fed-Fasted | WT, p=0.002 | KO, p=0.004 |
| *Glucose* | WT-KO | Fed, p=0.852 | Fasted, p=0.073 |
|  | Fed-Fasted | WT, p<0.001 | KO, p=0.028 |
| *Insulin* | WT-KO | Fed, p=0.071 | Fasted, p=0.780 |
|  | Fed-Fasted | WT, p=0.176 | KO, p=0.004 |
| *Ketones* | WT-KO | Fed, p=0.887 | Fasted, p=0.947 |
|  | Fed-Fasted | WT, p<0.001 | KO, p<0.001 |
| *Leptin* | WT-KO | Fed, p=0.061 | Fasted, p=0.873 |
|  | Fed-Fasted | WT, p=0.049 | KO, p=0.882 |
| *Corticosterone* | WT-KO | Fed, p=0.470 | Fasted, p=0.163 |
|  | Fed-Fasted | WT, p=0.002 | KO, p=0.005 |
