# Supplementary material for: The p75 neurotrophin receptor in AgRP neurons is necessary for homeostatic feeding and food anticipation
Source: eLife. 2020 Jan 29;9:e52623. doi: 10.7554/eLife.52623 (PMC7056271; doi:10.7554/eLife.52623)
Supplement: Table 2—source data 1. [file elife-52623-table2-data1.docx]

**Table 2- source data 1**

| ***p* values** | |  |  |
| --- | --- | --- | --- |
| **ZT16** |  |  |  |
| *Body Weight* | WT-KO | Fed, p<0.001 | Fasted, p=0.005 |
|  | Fed-Fasted | WT, p<0.001 | KO, p=0.547 |
| *Glucose* | WT-KO | Fed, p=0.690 | Fasted, p=0.619 |
|  | Fed-Fasted | WT, p<0.001 | KO, p<0.001 |
| *Insulin* | WT-KO | Fed, p=0.050 | Fasted, p=0.340 |
|  | Fed-Fasted | WT, p=0.003 | KO, p<0.001 |
| *Ketones* | WT-KO | Fed, p=0.326 | Fasted, p=0.274 |
|  | Fed-Fasted | WT, p<0.001 | KO, p<0.001 |
| *Leptin* | WT-KO | Fed, p=0.065 | Fasted, p=0.804 |
|  | Fed-Fasted | WT, p=0.013 | KO, p=0.006 |
| *Corticosterone* | WT-KO | Fed, p=0.064 | Fasted, p=0.175 |
|  | Fed-Fasted | WT, p=0.004 | KO, p=0.007 |
